# Jordan’s Population-Based Food Consumption Survey: Protocol for Design and Development

**DOI:** 10.2196/41636

**Published:** 2023-02-17

**Authors:** Islam Al-Shami, Huda Al Hourani, Buthaina Alkhatib, Omar Alboqai, Dima AlHalaika, Ayoub Al-Jawaldeh

**Affiliations:** 1 Department of Clinical Nutrition and Dietetics Faculty of Applied Medical Sciences The Hashemite University Zarqa Jordan; 2 Department of Nutrition and Food Science Faculty of Agriculture and Sciences Jerash University Jerash Jordan; 3 Regional Office for the Eastern Mediterranean World Health Organization Cairo Egypt

**Keywords:** household, food consumption, 24-hour dietary recall, children, adolescents, adults, older adults, overweight, obesity

## Abstract

**Background:**

One of the factors influencing health and well-being is dietary patterns. Data on food consumption are necessary for evaluating and developing community nutrition policies. Few studies on Jordanians’ food consumption and dietary habits at various ages have been conducted, despite the increased prevalence of overweight, obesity, and chronic diseases. This will be the first study focusing on Jordanians’ food consumption patterns that includes children, adolescents, adults, and older adults.

**Objective:**

This cross-sectional study aims to describe the design and methodology of the Jordan’s Population-based Food Consumption Survey, 2021-2022, which was developed to collect data on food consumption, including energy, nutrients, and food group intake, from a representative sample of Jordanians and to determine the prevalence of overweight and obesity and their relationship to food consumption.

**Methods:**

Participants were selected by stratified random sampling, using the *Estimated Population of the Kingdom by Governorate, Locality, Sex, and Households, 2020* as the sampling frame. The food consumption survey sample was at the population level, representing gender and age classes (8-85 years old). The data collection period was 6 months. Food consumption was assessed using 24-hour dietary recall (2 nonconsecutive days, 1 week apart) interviews representing weekdays and weekends. In addition to data on food consumption, information on the use of food supplements, sociodemographic and socioeconomic status, and health was gathered. Weight, height, and waist circumference were all measured.

**Results:**

The survey included 632 households with 2145 participants, of which 243 (11.3%) were children, 374 (17.4%) were adolescents, 1428 (66.6%) were adults, and 99 (4.6%) were older adults. Three food consumption databases were used to stratify the mean 24-hour dietary recall food consumption into energy intake, carbohydrates, proteins, fats, fiber, vitamins and minerals, and food groups. BMI was calculated and classified as normal, overweight, or obese. Central obesity was classified as normal or abnormal based on the waist-to-height ratio. The survey results will be disseminated based on age, energy, nutrient, and food group consumption. The prevalence of overweight and obesity by age group will be presented, as well as a comparison to the situation in Eastern Mediterranean countries.

**Conclusions:**

The survey data will be helpful in nutritional studies, assessing changes in dietary patterns, and developing and evaluating nutrition or health policies. It will be a solid base for developing a future national surveillance system on food consumption patterns with comprehensive food consumption, physical activity, biochemical, and blood pressure data.

**International Registered Report Identifier (IRRID):**

DERR1-10.2196/41636

## Introduction

The prevalence of noncommunicable diseases (NCDs) such as cardiovascular disease, high blood pressure, diabetes mellitus, and several forms of cancer has increased globally, as has obesity and overweight, and these trends have been linked to each other [1]. One of the major modifiable risk factors for obesity and NCDs is dietary patterns [2] and nutrient intake [3-6]. There has been a global movement in diet quality and nutrient intake toward a more Westernized pattern, which is characterized by a diet that is higher in added sugar, processed foods, fast food, and saturated fat while consuming fewer fruits, vegetables, dairy products, and fiber-rich foods [2,6,7]. This shift has been linked to the prevalence of obesity and NCDs being studied widely. To address these issues, various researchers have proposed studying general dietary patterns by looking at how foods and nutrients are consumed together as a solution. Numerous researchers have looked into the relationship between dietary habits and obesity. Thus, food consumption surveys are conducted worldwide [6,8].

In some countries, such as the United Kingdom [9] and the United States, continuous surveys are conducted over time [10]. At the same time, some surveys are conducted regularly (annually or biennial), such as in Germany [11], Italy [12], and Mexico [13]. Some surveys, on the other hand, are conducted irregularly, such as in France [14], Brazil [15], and Ireland [16]. Other surveys were carried out to assess dietary patterns, energy or nutrient intakes, or foods and beverages consumption in specific population subgroups or regions [17,18]. Food consumption surveys attempt to estimate the amount of food consumed by individuals. The outcomes vary from country to country and even within the same country due to differences in age, gender, and socioeconomic level.

The 24-hour dietary recall (24-h DR) is one of the most commonly used methods to assess food consumption. In some countries, all participants were subjected to multiple recalls [19,20]; however, in others, duplicate recalls were applied to a subsample of participants. The 24-h DR was carried out in a face-to-face or telephone interview using 24-h DR sheets or software to allow the structured and standardized collection of dietary intake data [21].

Other less common methods were used for estimating food consumption, including the food frequency questionnaire and dietary records [12,16].

Many countries use the results of dietary and nutrition surveys to monitor their population’s nutritional status, reveal trends in their health and dietary practices, determine links between food and health, identify populations at nutritional risk, and develop and implement nutritional policies tailored to reflect changes in dietary consumption [22-24].

The novelty of this study arises from the fact that it is the first to investigate the food consumption of different age groups in the Jordanian community, as well as obesity indices. Furthermore, the food consumption data were collected following the COVID-19 pandemic and its effects on food intake, body weight, and physical activity, primarily during school and university closures due to lockdowns. Therefore, this study aims to describe the design and methodology of Jordan’s Population-based Food Consumption Survey (JPFCS), 2021-2022, which was developed to collect dietary habits and nutritional status in a representative sample of the general Jordanian population.

The specific study objectives were as follows:

- To evaluate the energy and nutrient intake in the general Jordanian population, including children
- To examine the differences in food, energy, and nutrient intake among different subgroups of the population and to identify subgroups at risk for a deficient or excessive intake of specific foods or nutrients
- To determine the prevalence of overweight and obesity in the Jordanian population and the correlation with food and nutrient intakes

## Methods

### Design and Study Population

The JPFCS is a population-based cross-sectional study on household food consumption patterns that was conducted within all regions of the Hashemite Kingdom of Jordan between October 2021 and March 2022. Jordanians aged ≥8 years were defined as children, adolescents, adults, and older adults in the JPFCS study. Obtaining 24-h DR for children aged <8 years is difficult, especially out-of-home food intake (food eaten in schools, kindergarten, and daycare centers). Therefore, the data reliability is higher among children aged ≥8 years [22].

**Figure 1:**
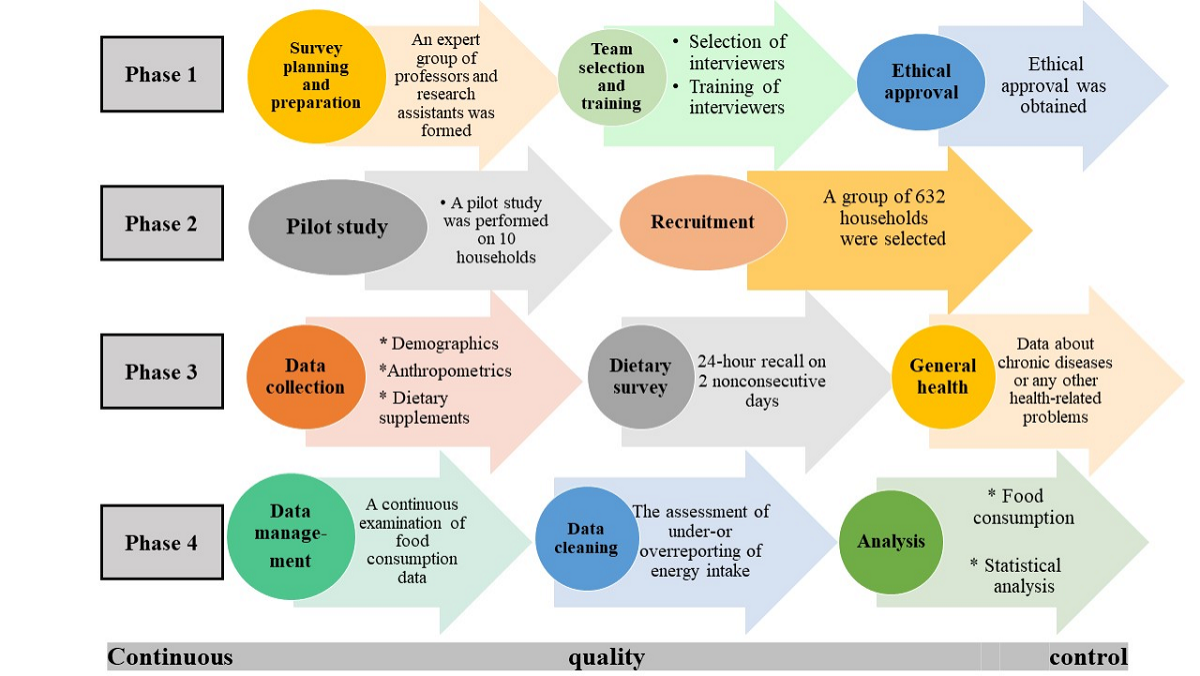
Flowchart of the study protocol.

This study included 4 phases. The first phase included survey planning, questionnaire preparation, interviewer selection and training, and obtaining ethical approval. The second phase involved conducting a pilot study and selecting households. The third phase included data collection from eligible household members on demographics, health, anthropometrics, and food consumption. The final phase will involve data management and analysis. The study protocol flowchart is presented in Figure 1.

### Sampling Frame

**Figure 2:**
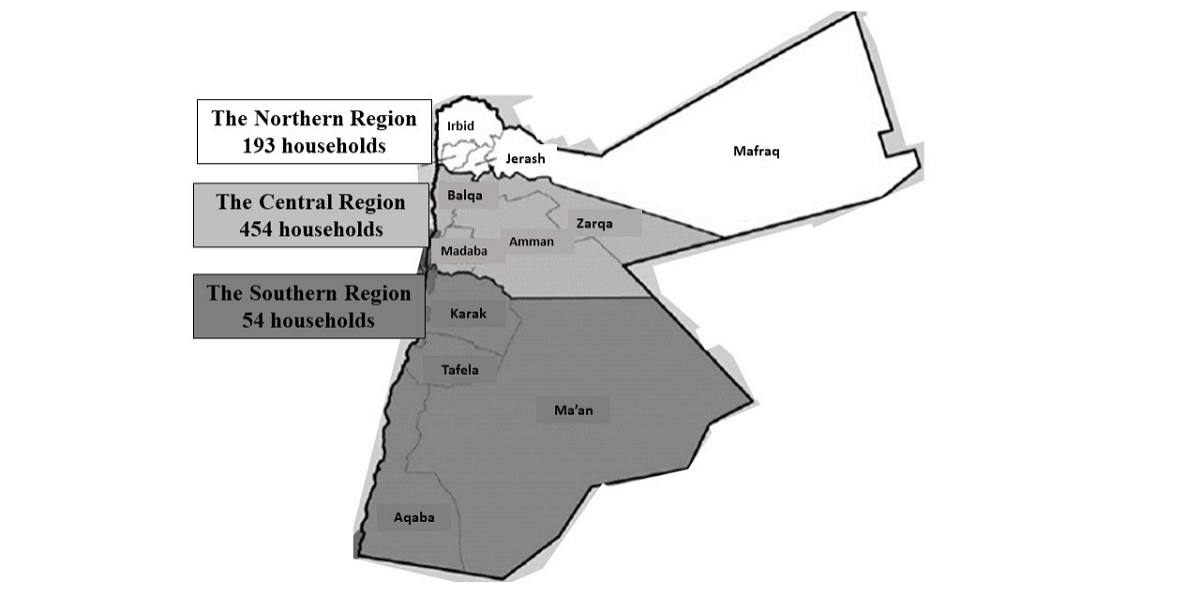
Map of regions and governorates in Jordan and the number of invited households.

Jordan covers 89,342 km^2^, with 10.8 million people in 2020 [25]. Geographically, the governorates of Jordan are located in 1 of 3 regions: the Central Region (Amman, Madaba, Balqa, and Zarqa), the Northern Region (Irbid, Mafraq, Ajloun, and Jarash), and the Southern Region (Aqaba, Karak, Ma’an, and Tafila). Each region has 4 governorates, including several districts and subdistricts, as shown in Figure 2. About 4.5 million people live in the Amman Governorate, almost half the country’s population.

As a sampling frame, a sample of Jordanian households was selected using the *Estimated Population of the Kingdom by Governorate, Locality, Sex, and Households, 2020* [25]. The sample size was calculated using the following formula:

n=N*X / (*X* + N – 1)

where N is the population size and

*X*=*Z*_α/2_^2^ × *p* × (1 – *p*) / *MOE*^2^

where Z_α/2_ is the critical value of the normal distribution at α/2 (eg, for a confidence level of 95%, α is .05, and the critical value is 1.96), *MOE* is the margin of error, *p* is the sample proportion.

A minimum sample size of 664 households was required. The number of governorate-selected households was calculated using the percentage of each region’s households in the overall number of Jordanian households, as shown in Table 1.

**Table 1:** Households distribution across regions.

Region	Households in each region (n=2,241,909), n (%)	Invited households, n (%)
Central	1,453,166 (64.8)	454 (64.8)
Northern	615,650 (27.5)	193 (27.5)
Southern	173,093 (7.7)	54 (7.7)
Total	2,241,909 (100)	701 (100)

### Informed Consent

Individuals who agreed to participate in the survey received detailed information about the survey objectives and what was expected from them; a disclosure of the survey’s requirements to preserve confidentiality, risks, and benefits; and a clear description of their decision to participate freely or not in the survey. After being presented with a detailed survey description, parents were asked for oral approval for their children to participate. No incentive money was offered for taking part in the survey. All data were recorded anonymously. Therefore, participants cannot be identified directly or through identifiers linked to the participants. At the start of the first home visit, participants or parents of children aged <12 years provided written informed consent.

### Ethics Approval

The Institutional Board Review committee at The Hashemite University reviewed and approved the survey protocol (No.7/13/2020/2021).

### Fieldwork Team: Management and Recruitment

During the project’s development, an expert group of professors and research assistants was formed, which appointed a primary coordinator, 3 co-coordinators, and a data entry specialist. The group was responsible for planning this survey domain, determining and developing the instruments, conducting the training, monitoring data collection, and planning the cleaning and organization processing of the data.

In all, 14 interviewers with nutrition backgrounds joined the group because of the need for a thorough knowledge of the available foods on the market and the recipes usually prepared within the study population. The population density determined the number of interviewers assigned to each region: 9 were assigned to the Central Region, 3 to the Northern Region, and 2 to the Southern Region.

### JPFCS Instruments

#### Structured Questionnaire and Data Management

A structured questionnaire was used to obtain information. The coding scheme of the participant’s questionnaire consisted of a 5-digit number that includes the governorate number, subgovernorate number, interviewer number, household number, and participant number.

After signing the consent form, a face-to-face interview with the participant obtained sociodemographic information on age, gender, number of household members, and monthly household income. General health data and intake of dietary supplements of the participants were also obtained. Questions on the presence of chronic diseases or other health-related problems were included.

Anthropometrics, including weight, height, and waist circumference, were measured. Interviews were conducted at the participant’s home at their convenience (usually outside working hours). Interviewers were encouraged to provide flexibility concerning appointment times. Additionally, the first 24-h DR was collected during these face-to-face interviews, whereas the second 24-h DR was collected by telephone.

#### Dietary Survey Tools

Dietary assessment was performed by the 24-h DR method on 2 nonconsecutive days (weekday and weekend). After respondents completed the questionnaire, the interviewer asked the respondent to recall and state the foods, beverages, and dietary supplements consumed in the preceding day, including their quantity, most commonly from midnight to midnight in the previous day. The interviewer obtained the first recall in a face-to-face interview with the eligible household member. The 24-h DR was typically completed in 20-40 minutes for each household member. The second recall representing the weekend was obtained by phone and showed acceptable estimates of the means and distributions of nutrient intakes among groups of individuals [26].

#### Food Description

All foods, beverages, and dietary supplements were recorded per food consumption occasion (morning, midmorning, noon, afternoon, evening, and late evening). As this was the collection of the first recall during face-to-face interviews, the date and day of the recall were recorded. In addition, all consumed foods or beverages; estimated amounts; methods of preparation; and brand names, if available, were documented. Interviewers used the multiple-pass method recommended by the United States Department of Agriculture. A 5-step dietary interview included multiple passes through the 24-h DR of the previous day, during which participants received cues to help them remember and describe the foods they consumed [27].

#### Portion Size Determination

Estimating portion sizes is an integral part of the dietary assessment. Various portion estimating aids have been developed, such as household measurement items (eg, standard measuring glasses, cups, teaspoons, and tablespoons), food models, food portions (obtained from fact sheets and manufacturer’s information presented on the product’s containers), food pictures, and other visual aids that may be used to help the participants judge and report portion size.

Participants in this survey were assisted in describing the actual quantity of food or beverage consumed by giving them a well-known idea about the quantity by using a colored food atlas containing over 100 foods, and composite recipes comprising photos of various foods and meals regularly consumed in Jordanian diets were created. For each food item, at least four photographs showed portion sizes. However, to our knowledge, no comprehensive food atlas has been developed in Jordan.

The photographs were categorized into different groups: bread and grains, fruits and vegetables, milk and dairy products, meat and meat alternatives, sweets and desserts, fat, and composite dishes. In addition, photographs of standard measurement devices in the Jordanian diet, such as cups, teaspoons, and tablespoons, were included.

#### Dietary Supplements

This section highlights the usage of dietary supplements: the frequency and type of dietary supplements. Usually, the use of dietary supplements was recovered by 2 methods. The first method used the 24-h DR; dietary supplements were recorded per consumption occasion and quantified and described as consumed [28]. The second method asked the frequency of use, in the last 12 months, of a predefined list of different dietary supplement types (eg, supplements of vitamins, such as D and folate; supplements of minerals, such as calcium and iron; supplements of multivitamins; and supplements of fatty acids, herbs, plants, and probiotics) [28].

#### Dietary Database

The detailed food consumption data were entered into a food composition database. Three food composition databases were used: ESHA’s Food Processor nutrition analysis software (version 11:0; ESHA Research); *Composition of Local Jordanian Food Dishes* [29]; and *Lebanon Food Composition Data: Traditional Dishes, Arabic Sweets, and Market Foods* [30]. The final dietary variables that will be generated will represent the average of 2 recalls and be used for further analyses. Data about the total daily energy intake, macronutrients, vitamins, and minerals will be evaluated for all age groups.

#### Training of Interviewers

Smaller-group interviewers received 1-day training from the subnational coordinators and Zoom training (Zoom Video Communications) upon need. The training content included general study information, study objectives, instructions on the interviewer’s attitudes, coding scheme, procedures for completion of the 24-h DR, and the use of the colored photographic food atlas. Interviewers were provided with the protocol for home visits and telephone calls, the content of the questionnaires, and the anthropometric measurement procedure. New interviewers recruited during the fieldwork received the same training except individually. In total, the training was repeated at least five times.

#### Anthropometrics

Anthropometric measurements, including height (cm), weight (kg), and waist circumference (cm), were performed on children and adults according to standard procedures by a trained nutritionist. Height was measured to the nearest centimeter, with participants in a standing position with light clothing and barefoot, using a portable wall stadiometer. Body weight was measured in the same conditions to the nearest tenth of a kilogram using a digital scale (Microlife WS 50). Waist circumference was measured using an anthropometric tape at the narrowest point between the lower costal border and the iliac crest. BMI (kg/m^2^) was calculated from the height and weight measurements according to the Quetelet formula [22]: *BMI = weight (kg) / height (m^2^)*, and the waist-to-height ratio was calculated by dividing the participant’s waist circumference by his or her height [22].

### Quality Controls

The following quality control steps were taken before, during, and after data collection:

1. A pilot study was performed for 10 households on the tools of the study to enhance the quality of the collected information.
2. Interviewers were chosen for their knowledge of 24-h DR interviews, market foods, traditional recipes, and anthropometric measurements. Furthermore, those having a solid background in the assessment of nutritional status, both theoretical and practical, and having received adequate training to conduct anthropometric measurements and assess food intake in various ways while studying at the university were chosen.
3. Training of the interviewers before data collection via face-to-face and Zoom meetings
4. A WhatsApp (Meta Platforms) group was formed to facilitate communication between the national coordinator, subnational coordinators, data entry specialists, and interviewers. It responds to and clarifies any study-related issue.
5. A continuous examination of food consumption data as they are entered into the nutrition analysis software
6. As an essential issue to consider, the assessment of under- or overreporting of energy intake was carried out using the cutoff points and method proposed by Goldberg et al [31] and Black [32].

### Statistical Analysis

Data analysis was performed using SPSS software (version 22.0; IBM Corp). The normality of the distributions will be assessed through the Kolmogorov-Smirnov test and kurtosis and skewness values. Continuous variables will be presented as mean values and standard deviations, percentages for numerical variables, and frequencies (n and %) for categorical variables. This analysis will be stratified by sex and age.

Inferential statistics such as the Student *t* test (2-tailed), chi-square test, and independent samples *t* test (2-tailed) will be used based on the sample distribution and test statistics. Statistical significance will be accepted at P<.05, with a 95% confidence level.

## Results

### Sample Size

**Figure 3:**
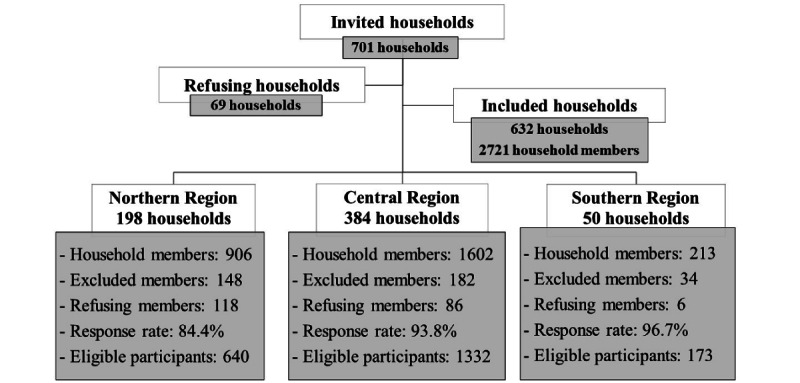
Hierarchical chart for household sampling, participant distribution, the number of excluded individuals, response rates, and refusals by region.

A representative sample of 701 households was randomly invited, and 632 households were included in the study, with a 90.2% response rate. A total of 2721 household members were approached, with a response rate of 78.8% (n=2145). Female subjects who were pregnant or lactating and children aged <8 years in the selected households were excluded from the study. The overall refusal rate of the included household members was 7.7% (210/2721). The hierarchical chart for household sampling, participant distribution, the number of excluded individuals, response rates, and refusals by region is shown in Figure 3.

The age groups for eligible male and female household members were as follows: 8-12, 13-19, 20-64, and ≥65 years. Table 2 summarizes the number of subjects interviewed for each sex of children, adolescents, adults, and older adults.

The 2145 subjects were divided into 4 age groups: 11.3% (n=243) were children (8-12 years), 17.4% (n=374) were adolescents (13-19 years), 66.6% (n=1428) were adults (20-64 years), and 4.6% (n=99) were older adults (≥65 years; Table 2).

The data collected included weight, height, and waist circumference measurements; the response rate for those measurements was high, with the lowest response rate (117/119, 98.3%) for waist circumference in girls aged 8-12 years. The collection of the second 24-h DR for dietary assessment revealed the lowest response rate (114/124, 91.9%) in boys aged 8-12 years (Table 3).

Before estimating nutrient and food group intakes, participants will be divided into 3 groups based on energy intake: underreporters, plausible reporters, and overreporters; plausible reporters will only be considered when estimating nutrient and food group intakes. The survey results will be disseminated in national and international scientific journals.

**Table 2:** Distribution of participants by sex and age group.

Age group	Total (n=2145), n (%)	Male (n=945, 44.1%), n (%)	Female (n=1200, 55.9%), n (%)
Children (8-12 years)	243 (11.3)	124 (13.1)	119 (9.9)
Adolescents (13-19 years)	374 (17.4)	163 (17.2)	211 (17.6)
Adults (20-64 years)	1428 (66.6)	608 (64.3)	820 (68.4)
Older adults (≥65years)	99 (4.6)	50 (5.3)	49 (4.1)

**Table 3:** Response rates of the collected data by gender and age group.

Collected data	Children			Adolescents			Adults			Older adults	
	Boys (n=124), n (%)	Girls (n=119), n (%)	Boys (n=163), n (%)		Girls (n=211), n (%)	Men (n=608), n (%)		Women (n=820), n (%)	Men (n=50), n (%)		Women (n=49), n (%)
Weight measurement	124 (100)	119 (100)	163 (100)		211 (100)	608 (100)		818 (99.8)	50 (100)		49 (100)
Height measurement	124 (100)	118 (99.2)	163 (100)		211 (100)	608 (100)		81 (99.8)	50 (100)		49 (100)
WC^a^ measurement	124 (100)	117 (98.3)	163 (100)		211 (100)	608 (100)		817 (99.6)	50 100)		49 (100)
First 24-h DR^b^	123 (99.2)	119 (100)	163 (100)		210 (99.5)	608 (100)		818 (99.8)	50 (100)		49 (100)
Second 24-h DR	114 (91.9)	115 (96.6)	157 (96.3)		206 (97.6)	601 (98.8)		808 (98.5)	50 (100)		48 (98.0)
Both 24-h DRs combined	114 (91.9)	115 (96.6)	157 (96.3)		206 (97.6)	601 (98.8)		808 (98.5)	50 (100)		48 (98.0)

^a^WC: waist circumference.

^b^24-h DR: 24-hour dietary recall.

## Discussion

### Expected Findings

The study aimed to describe the design and methodology of a population-based food consumption survey to evaluate energy, nutrient, and food group intake in various age groups and the relationship with the prevalence of overweight and obesity.

Food consumption patterns in Jordan have not been studied at the population level. The published studies focused on specific age groups, such as food consumption among university students [33,34] and food and nutrient consumption in certain disease cases [35,36]. These studies used a food frequency questionnaire to focus on food group consumption, whereas 24-h DR was rarely used. Some of the studies mentioned above were pilot studies or had small sample sizes. Furthermore, food consumption data for various age groups and households are still scarce in many Middle Eastern countries.

The total number of respondents in this study was 2145, divided into 4 age groups: children, adolescents, adults, and older adults. The findings of this study will concentrate on food consumption patterns, food group consumption, macronutrient consumption, and some micronutrient consumption, as well as anthropometric indices of obesity such as BMI and weight-to-height ratio in the studied age groups. In line with this protocol, Bel and de Ridder [37] completed a national survey on the Belgian national food consumption data collection in 2200 individuals (aged 10-64 years). Food intake data were collected with 2 nonconsecutive 24-h DRs.

Data from national studies will assist governments and international organizations in supplying statistical elements that will eventually advise national social security and health policies [38]. Additionally, this population-based survey will shed light on the whole population’s energy intake, risk assessment, and nutritional status studies, with essential suggestions for developing policies [37,38].

### Strengths and Limitations

Among study limitations, the household sample size was smaller than recommended; biochemical data, blood pressure measurements, food safety, hygiene, and lifestyle pattern were not included. Moreover, it was challenging to collect data about food consumption for children aged <8 years. However, among the study’s strengths is that the protocol was similar to other population-based dietary surveys. Participants in the survey were from both sexes and were distributed in all representative age groups in Jordan. Additionally, the distribution of interviews on weekdays and seasonality was confirmed. The core strength of this protocol is that the dietary assessment was completed following the standards of the Jordanian diet and Arabic regions that covers food and nutrient intake, nutritional status, and eating habits. Furthermore, precise and comprehensive food consumption data are crucial for the food consumption assessment based on the food atlas prepared and introduced by the team workers.

### Future Perspectives

As the first population-based household survey, it will provide updated information about dietary habits and food consumption patterns for various age groups, especially because it was conducted following the COVID-19 pandemic. The findings serve as a descriptive baseline for future follow-up and a critical starting point for future food consumption pattern studies of some Jordanian age groups.

### Conclusions

This population-based food consumption survey aims to collect data on the food consumption of a sample representing various age groups. It will provide the most comprehensive evaluation of food consumption, identify the components of consumed food and their nutrient contents, examine energy and nutrient intakes, and identify the prevalence of overweight and obesity in the Jordanian community. It will serve as a solid nutritional background for future studies, help assess dietary patterns changes, develop and evaluate nutrition policies, and serve as a solid foundation for developing a future national surveillance system on food consumption patterns.

## Data Availability

The data sets generated during and/or analyzed during the current study are available from the corresponding author upon reasonable request**.**

## Abbreviations

JPFCS: Jordan’s Population-based Food Consumption Survey
NCD: noncommunicable disease
24-h DR: 24-hour dietary recall
